# Design of a High Coupling SAW Resonator Based on an Al/41° Y-X LiNbO_3_/SiO_2_/poly-Si/Si Structure for Wideband Filter

**DOI:** 10.3390/mi16030323

**Published:** 2025-03-11

**Authors:** Xiaoyu Wang, Yang Chang, Qiaozhen Zhang, Luyao Liu, Xinyi Wang, Haodong Wu

**Affiliations:** 1 School of Electronic Information Engineering/School of Integrated Circuits, Nanjing Vocational University of Industry Technology, Nanjing 210023, China; niitwxy@163.com; 2 College of Information, Mechanical and Electrical Engineering, Shanghai Normal University, Shanghai 200233, China; 1000484226@smail.shnu.edu.cn (Y.C.); 1000466420@smail.shnu.edu.cn (L.L.); wangxinyi@shnu.edu.cn (X.W.); 3 Key Laboratory of Modern Acoustics, Ministry of Education, Department of Acoustic Science and Engineering, School of Physics, Nanjing University, Nanjing 210093, China; haodongwu@163.com

**Keywords:** surface acoustic wave, acoustic resonator, high coupling, multi-layer film, wideband filter

## Abstract

With the rapid development of fifth-generation (5G) mobile communication technology, the performance requirements for radio frequency front-end surface acoustic wave (SAW) devices have become increasingly stringent. Surface acoustic wave devices on piezoelectric thin film-based layered structures with high electromechanical coupling coefficients and low-frequency temperature compensation characteristics have emerged as a key solution. In this work, a SAW resonator based on an Al/41° Y-X LiNbO_3_/SiO_2_/poly-Si/Si multi-layered structure is proposed. FEM modeling of the proposed resonator and the influences of the thicknesses of the LiNbO_3_, SiO_2_, and Al electrodes on performances such as the parasitic noise, bandwidth, and electromechanical coupling coefficient are analyzed. Optimal parameters for the multi-layer piezoelectric structure are identified for offering large coupling up to 24%. Based on these findings, a single-port SAW resonator with an Al/41° Y-X LiNbO_3_/SiO_2_/poly-Si/Si substrate structure is fabricated. The experimental results align well with the simulation results; meanwhile, the SAW filter based on the proposed resonator demonstrates that a center frequency of 2.3 GHz, a 3-dB fractional bandwidth of 23.48%, and a minimum in-band insertion loss of only 0.343 dB are simultaneously achieved. This study provides guidance for the development of multi-layer film SAW resonator-based filters with high-performance.

## 1. Introduction

Surface acoustic wave filters utilize acoustic waves that propagate on the surface of piezoelectric crystals to provide excellent frequency selectivity with a compact device size [1,2,3]. As mobile communication technology advances, the fifth-generation has become the leading force in current and future wireless communications. As critical components in 5G systems, SAW filters are crucial for achieving high-speed data transmission, low loss, and wide bandwidths [4,5,6,7]. Their application in 5G systems enhances data transfer rates, reduces latency, and improves signal coverage, significantly optimizing system performance and communication quality. Traditional SAW devices typically use bulk piezoelectric single crystals such as LiNbO_3_, quartz, and LiTaO_3_, which, despite their lower acoustic velocities, ionic volatility at high temperatures, electromechanical coupling coefficient, and quality factors, are limited in high-frequency applications [8,9,10]. Although ZnO and AlN thin films offer higher acoustic velocities, their lower electromechanical coupling coefficients make them primarily suitable for narrowband SAW devices [11,12].

To address these limitations, researchers have explored bonding piezoelectric single-crystal films with other materials to leverage their combined advantages and develop high-performance SAW devices. The selection of appropriate materials can result in high sound propagation speeds, high electromechanical coupling coefficients, and improved TCF characteristics. For instance, Hashimoto et al. investigated the acoustic wave propagation characteristics of Cu-Grating/Y-X LiNbO_3_/SiO_2_/Si substrates [13]. Ikata et al. developed trapezoidal SAW filters for different frequencies and demonstrated the potential of this type of substrate structure in the development of ultra-wideband and low-loss RF SAW filters [14]. Tao et al. studied the effects of various material thicknesses on the performance of SAW filters based on LiNbO_3_/SiO_2_/Si multi-layer structures and experimentally validated their theoretical results [15]. Hus et al. designed wideband radio-frequency shear-horizontal surface acoustic wave resonators based on a LiNbO_3_/SiO_2_/Si functional substrates and demonstrated the feasibility of developing broadband acoustic RF devices for potential 5G wireless communications [16]. Feng et al. examined a SAW filter based on IDT (Cu)/15° Y-X LiNbO_3_/SiO_2_/Si substrate structures and analyzed the impact of electrode parameters on the electromechanical coupling coefficient and the performance of the trapezoidal filter [17]. Their results revealed excellent bandpass filter characteristics, a good frequency temperature coefficient, and strong power durability. Pan et al. explored the performance of a SAW resonator on a 42° Y-X LiTaO_3_ multi-layer substrate, optimized the thickness of each layer in the 42° Y-X LiTaO_3_/SiO_2_ substrate, and studied the effects of device structure parameters on dispersion and slow-wave curves. Their experimental results were consistent with simulation results, indicating that optimizing the materials and thicknesses of multi-layer film structures can significantly enhance SAW device performance [18].

This paper proposes a SAW resonator based on a 41° Y-X LiNbO_3_/SiO_2_/poly-Si/Si multi-layered structure. A comprehensive analysis of the proposed SAW resonator is conducted using analytical theory and finite element methods. First, the governing equations for the SAW propagating along the multi-layered structure are derived based on the constitutive relationships between the mechanical displacement and electric field in the piezoelectric film. Subsequently, finite element software COMSOL5.5 is utilized to investigate the effects of LiNbO_3_, SiO_2_, and Al electrodes on key performance parameters, such as the device admittance characteristics, phase velocity, and bandwidth, providing guidance for the comprehensive optimal design of SAW devices. Finally, based on the simulation results, the optimal substrate and electrode structures are determined. A SAW resonator is fabricated on a 41° Y-X LiNbO_3_/SiO_2_/poly-Si/Si multi-layered substrate and its application to a wideband SAW filter is explored.

## 2. Theoretical Background

Piezoelectric materials are anisotropic dielectrics that experience polarization changes under external forces in what is known as the piezoelectric effect. This effect reflects the coupling between mechanical and electrical quantities. Apart from elastic strain and stress fields, there are also interactions between electric fields and electric displacement fields. The discussion here is confined to the linear range, where one mechanical quantity and one electrical quantity are considered as variables. Therefore, the strain and electric field strength are used as variables, and constitutive equations for the stress and electric displacement are derived based on Hooke’s law and electrical relationships. The stress and electric displacement are expressed in tensor form as [19,20]:

(1) $$T_{I}=c_{IJ}^{E}S_{J}-e_{Ij}E_{j}$$

(2) $$D_{i}=e_{iJ}S_{J}+\varepsilon_{ij}^{S}E_{j}$$

Here, ***T****_I_*, ***D****_i_*, *c_IJ_^E^*, *e_iJ_*, and *ε_ij_^S^* are the stress, electric displacement, material elastic constant, piezoelectric stress constant, and dielectric constant, respectively.

According to Hooke’s law, the relationship between the strain ***S*** and displacement ***u*** is:

(3) $$S={\nabla_{s}}^{\prime }u$$

**Figure 1:**
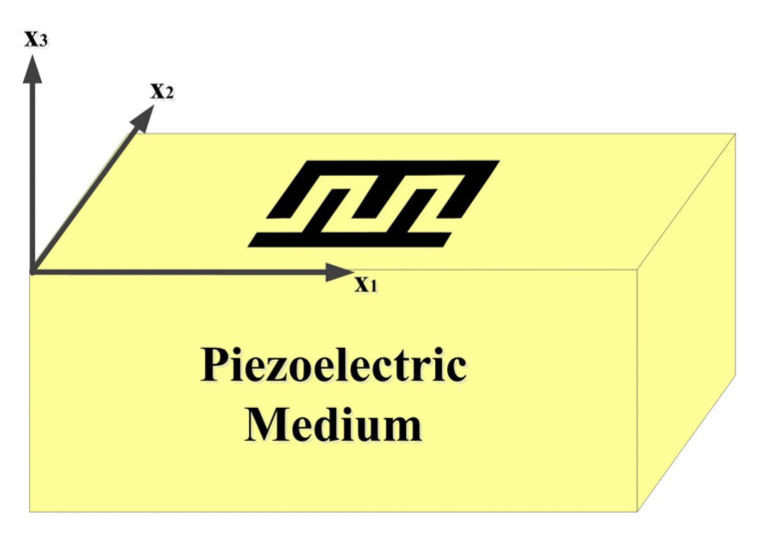
The coordinate system of SAW propagation on a 41° Y-X LiNbO_3_/SiO_2_/poly-Si/Si multi-layer film structure.

According to Figure 1:

(4) $$\nabla_{s}=\left[\begin{array}{cccccc} \frac{\partial }{\partial x_{1}} & 0 & 0 & 0 & \frac{\partial }{\partial x_{3}} & \frac{\partial }{\partial x_{2}}\\ 0 & \frac{\partial }{\partial x_{2}} & 0 & \frac{\partial }{\partial x_{3}} & 0 & \frac{\partial }{\partial x_{1}}\\ 0 & 0 & \frac{\partial }{\partial x_{3}} & \frac{\partial }{\partial x_{2}} & \frac{\partial }{\partial x_{1}} & 0 \end{array}\right]$$

In electrical theory, the relationship between the electric displacement ***D*** and charge density *ρ*, and the electric field strength ***E*** and potential *ϕ* is:

(5) E=−∇ϕ

The piezoelectric equilibrium equations for SAW devices can be obtained based on Newton’s second law and Maxwell’s equations, expressed in tensor form:

(6) $$\nabla_{s}C\nabla_{s}\prime u+\nabla_{s}e\prime \nabla \phi =\rho \ddot{u}$$

(7) $$-\nabla \cdot \varepsilon \nabla \phi +\nabla \cdot e\nabla_{s}^{\prime }u=0$$

## 3. Modeling and Simulation

**Figure 2:**
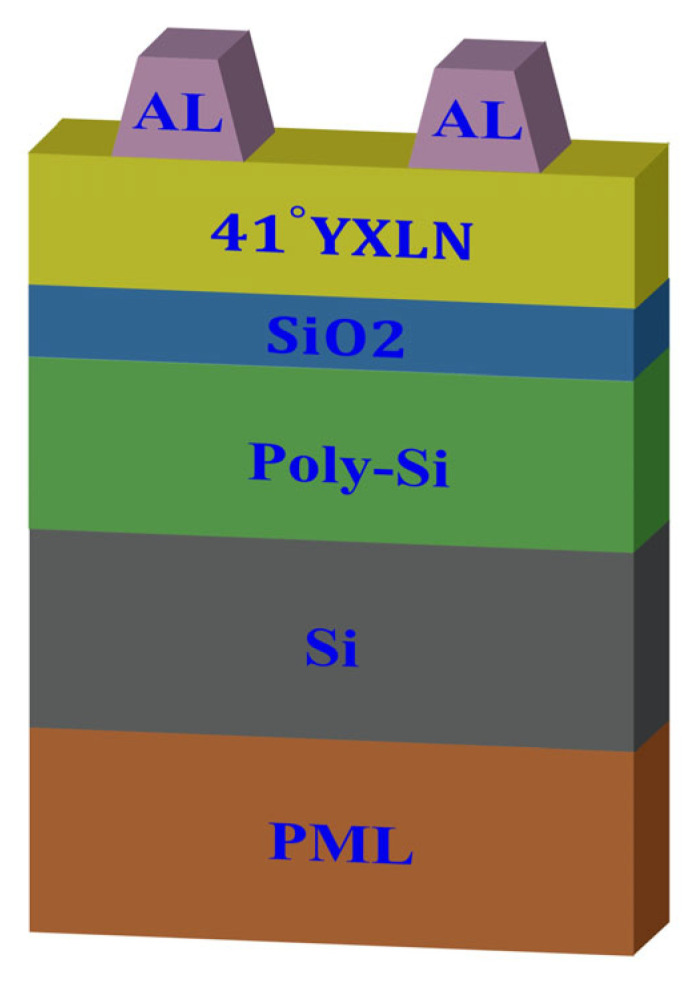
A schematic diagram of the periodic FEM model of the resonator on a 41° Y-X LiNbO_3_/SiO_2_/poly-Si/Si multi-layer film structure.

**Figure 3:**
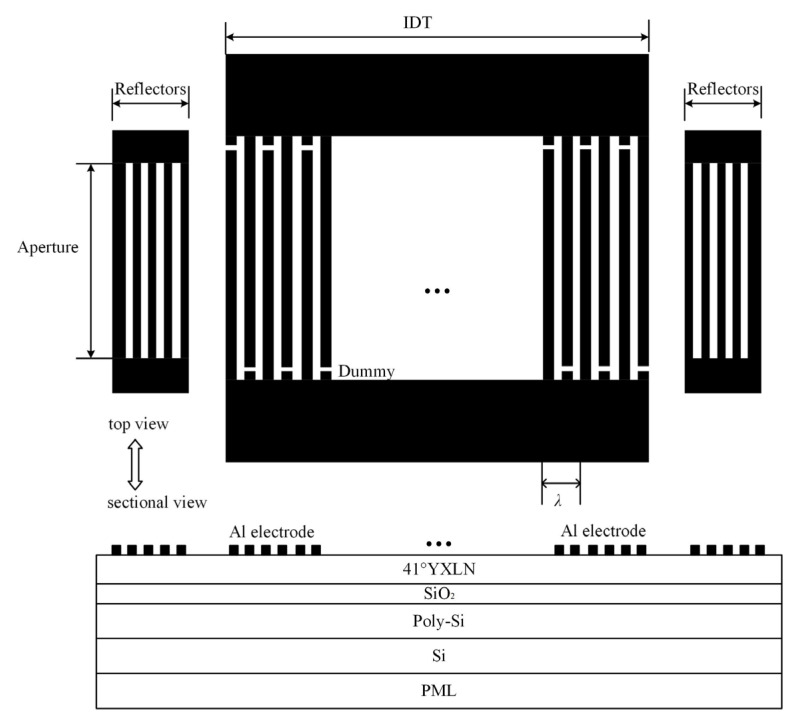
A schematic diagram of the one-port SAW resonator on a 41° Y-X LiNbO_3_/SiO_2_/poly-Si/Si multi-layer film structure.

The high performance of multi-layer SAW devices is achieved by utilizing the advantages of various materials and multi-layer film structures to confine the acoustic wave energy to the material’s surface and enhance the quality factor. This paper comprehensively analyzes the effects of different material thicknesses on the performance of SAW resonators based on a 41° Y-X LiNiO_3_ substrate. The SAW resonator structure is illustrated in Figure 2 and includes a perfectly matched layer (PML), Si, poly-Si, SiO_2_, 41° Y-X LiNiO_3_, and an Al electrode. The SiO_2_ serves as both a low acoustic impedance layer and a temperature compensation layer, the poly-Si acts as an electron trapping layer, and the high-resistivity Si serves as the supporting substrate. Figure 3 is the schematic diagram of the one-port SAW resonator on a 41° Y-X LiNbO_3_/SiO_2_/poly-Si/Si multi-layer film structure.

Assuming the SAW propagates along a designated direction in the multi-layer film structure, the full-scale 3D finite element model is simplified to a double-finger structure with a single-period size. A PML is also constructed at the bottom to absorb acoustic waves propagating into the substrate, eliminating interference from reflected signals. Periodic boundary conditions are applied on both sides of the model to extend it infinitely in the propagation direction. To ensure both simulation accuracy and speed, the mesh size in the area below the electrode is set to be smaller than in the other substrate areas. This is because the geometric shape of the Al electrodes in practical processing is trapezoidal, and the acoustic wave energy in the thickness direction decays rapidly, concentrating primarily on the surface of the piezoelectric film.

**Figure 4:**
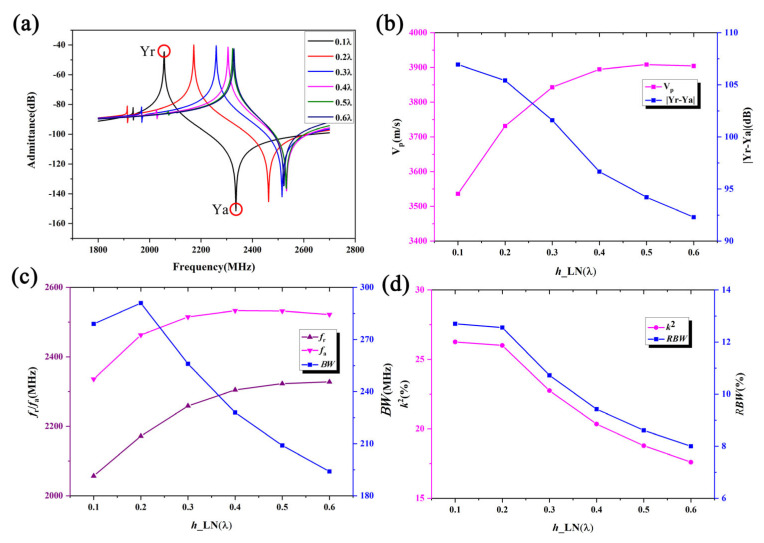
The simulation results for the SH wave with different LiNbO_3_ thicknesses. Panels (**a**–**d**) show the admittance, V_p_, resonant/anti-resonant frequency, and electromechanical coupling coefficient/relative bandwidth, respectively.

The COMSOL finite element software can simulate various physical fields, such as vibrations and electric fields [21]. Thus, as shown in Figure 2, an infinite-period model is used for frequency-domain simulation with the MUMPS solver to optimize the thicknesses of different layers for optimal device performance. Figure 4 shows the frequency characteristics as a function of the LiNbO_3_ thickness *h*_LN. The IDT period is set to 1.61 μm, the IDT metalization ratio is 0.5, the Al electrode thickness *h*_Al is 180 nm, the SiO_2_ thickness *h*_SiO_2_ is 450 nm, and the poly-Si thickness is fixed at 1 μm. During the calculations, the thickness of the bottom Si substrate is set to 4.83 μm, and the PML thickness is set to 3.22 μm. The corresponding material parameters of the simulation are show in Table 1. Figure 4a compares the admittance curves of the SAW resonator for different LiNbO_3_ thicknesses. Here, Y_r_ and Y_a_ are the peak value of the admittance curve. It is evident that the LiNbO_3_ thickness significantly impacts the frequency characteristics of the SAW resonator, with the resonant frequency increasing as the LiNbO_3_ thickness increases. Figure 4a shows that spurious waves near the resonant frequency are effectively suppressed. Figure 4b clearly illustrates the dependence of the phase velocity V_p_ and admittance difference |Y_r_ − Y_a_| on the LiNbO_3_ thickness. The phase velocity increases with the LiNbO_3_ thickness, indicating dispersion effects for high-frequency SAWs, with high-speed waves appearing within the expected range. Conversely, the admittance difference decreases with increasing LiNbO_3_ thickness, with a smaller admittance difference indicating a poorer out-of-band suppression capability. Figure 4c shows the effects of the LiNbO_3_ thickness on the resonant frequency *f_r_* which is the frequency corresponding to Y_r_, anti-resonant frequency *f_a_* which is the frequency corresponding to Y_a_, and bandwidth *BW*. It is observed that *f_r_* and *f_a_* both increase with the LiNbO_3_ thickness, while *BW* initially increases and then decreases, reaching a maximum value when the LiNbO_3_ thickness is 0.2 L. Figure 4d shows that the electromechanical coupling coefficient *k*^2^ and relative bandwidth *RBW* values decrease non-linearly with increasing LN thickness. Here, V_p_, *BW*, *k*^2^, and *RBW* could be describe as:

(8) $$V_{p}=\frac{(f_{r}+f_{a})\lambda }{2}$$

(9) $$BW=f_{a}-f_{r}$$

(10) $$RBW=\frac{2(f_{a}-f_{r})}{f_{a}+f_{r}}\%$$

(11) $$k^{2}=\frac{\Delta f}{f_{a}}*{\left(\frac{\pi }{4}\right)}^{2}=\frac{f_{a}-f_{r}}{f_{a}}*{\left(\frac{\pi }{4}\right)}^{2}$$

With an increased LiNbO_3_ thickness, parameters such as the resonance frequency, phase velocity, admittance deviation, electromechanical coupling coefficient, and bandwidth change monotonically. This is because the energy of the SH wave is primarily concentrated on the material’s surface. As the piezoelectric film becomes thicker, its performance becomes similar to that of an SH wave on a 41° YX LiNbO_3_ structure.

**Figure 5:**
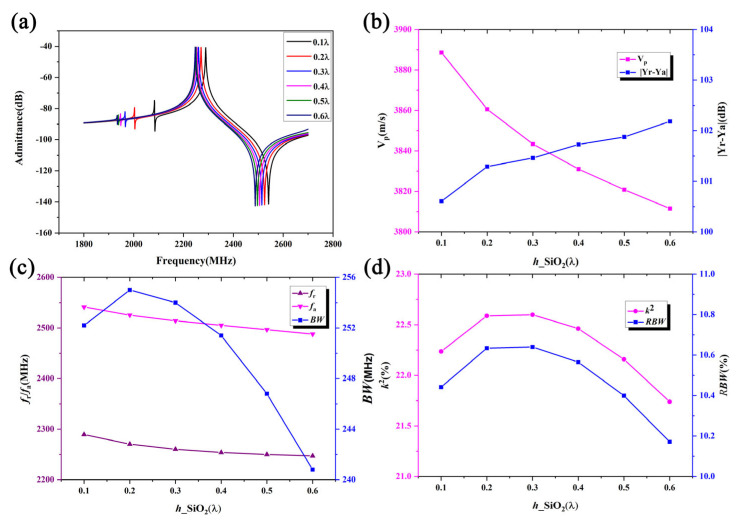
The simulation results of the SH wave for different SiO_2_ thicknesses. Panels (**a**–**d**) show the admittance, V_p_, resonant/anti-resonant frequency, and electromechanical coupling coefficient/relative bandwidth, respectively.

The effect of the SiO_2_ layer, which provides temperature compensation, on the overall performance of the surface acoustic wave resonator is also noteworthy. To study this, the IDT period is also set to 1.61 μm, with the IDT metalization ratio is the same as above, an Al electrode thickness of 180 nm, a LiNbO_3_ thickness of 490 nm, and the poly-Si thickness fixed at 1 μm. Figure 5a compares the admittance curves of the SAW resonator with different SiO_2_ thicknesses. It can be seen that the resonance frequency decreases monotonically with increasing SiO_2_ thickness. Figure 5b shows the relationship between the phase velocity and admittance deviation as a function of the SiO_2_ thickness. It is clear that the phase velocity decreases with increasing SiO_2_ thickness while the admittance deviation increases. Figure 5c shows the effect of the SiO_2_ thickness on the resonance frequency, anti-resonance frequency, and bandwidth. It can be observed that the resonance frequency and the anti-resonance frequency decrease slowly with increasing SiO_2_ thickness, and the bandwidth first increases and then gradually decreases, reaching a maximum value when the SiO_2_ thickness is 0.2 L. Figure 5d shows the relationship between *k*^2^ and relative bandwidth *RBW* as a function of the SiO_2_ thickness. Clearly, both the electromechanical coupling coefficient *k*^2^ and *RBW* values decrease non-linearly with increasing SiO_2_ thickness, reaching a maximum value at SiO_2_ thicknesses of approximately 0.2 λ to 0.3 λ.

**Figure 6:**
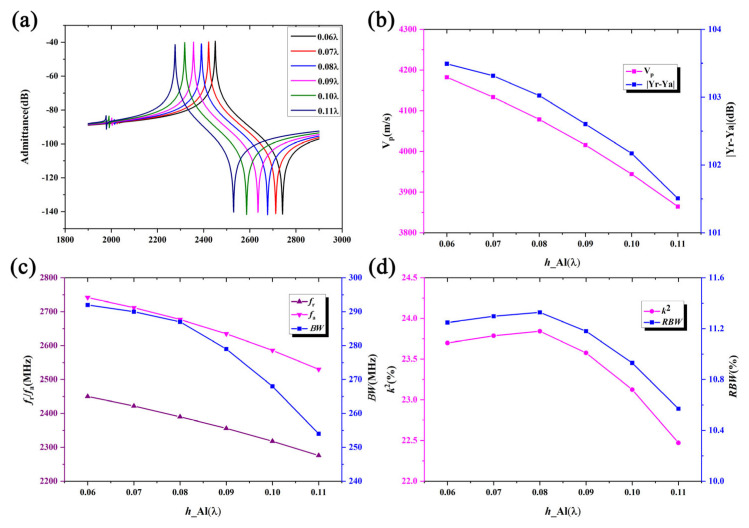
The simulation results of the SH wave with different Al electrode thicknesses. Panels (**a**–**d**) show the admittance, V_p_, resonant/anti-resonant frequency, and electromechanical coupling coefficient/relative bandwidth, respectively.

Apart from LiNbO_3_ and SiO_2_, the thickness of the Al electrode also affects the SAW performance due to mass loading. Therefore, the effect of the Al electrode thickness is also investigated in this work. Specifically, the IDT period is set to 1.61 μm, with a metal duty cycle of 0.5, a LiNbO_3_ thickness of 490 nm, a SiO_2_ thickness of 420 nm, and a poly-Si thickness of 1 μm. The performance of the SAW resonator with different Al electrode thicknesses is calculated. Figure 6 shows the change in the admittance curves with increasing Al thickness. It is clear that as the Al thickness increases, the mass loading of the Al electrode causes the resonance frequency to decrease gradually. Additionally, since different acoustic wave modes in the piezoelectric dielectric have different excitation efficiencies, it is necessary to select an appropriate Al electrode thickness to suppress spurious waves within the passband. Figure 6 shows that as the Al electrode thickness varies, the phase velocity, resonance frequency, *k*^2^, and *RBW* values exhibit similar curves to those seen for a changing SiO_2_ thickness. However, in contrast to the SiO_2_ thickness variation curves, the admittance deviation and bandwidth decrease with increasing Al electrode thickness.

The above analysis shows that the LiNbO_3_ thickness significantly impacts the velocity, admittance deviation, and *k*^2^ values. Compared to the LiNbO_3_ thickness, the SiO_2_ thickness has a smaller effect on these parameters, and the Al electrode thickness primarily affects the velocity. Optimal resonator performance with high coupling of 24% operating frequency more than 2.2 GHz is achieved when configured with a LiNbO_3_ thickness of 490 nm, a SiO_2_ thickness of 420 nm, and an Al electrode thickness of 130 nm.

## 4. Design Verification of Resonator and Its Application to Filter

Based on the above simulation results, a multi-layer Al/41° Y-X LiNbO_3_/SiO_2_/poly-Si/Si structure SAW resonator was manufactured as shown in Figure 3, with an Al electrode thickness of 130 nm, a LiNbO_3_ thickness of 490 nm, a SiO_2_ thickness of 420 nm, and a polysilicon thickness of 1300 nm. The resonator includes 251 IDTs and 5 finger reflector arrays, and its aperture is weighted using a cosine function in order to achieve lateral mode suppression.

**Figure 7:**
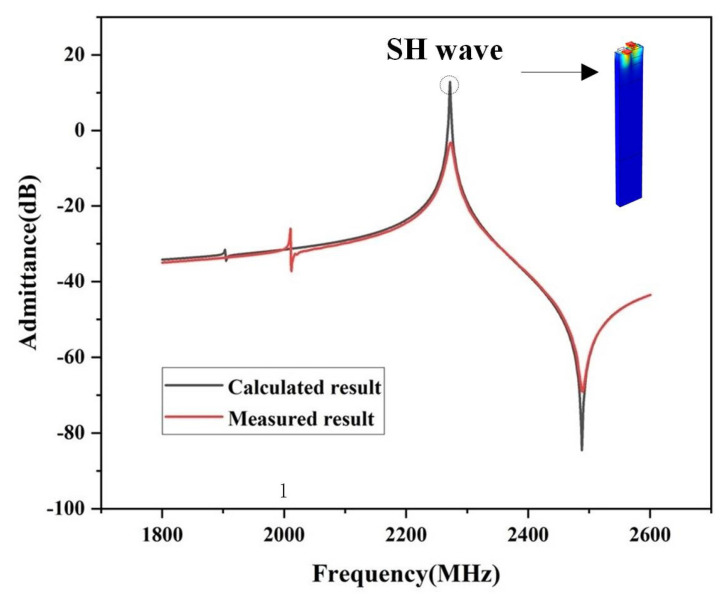
The calculated and measured frequency response results of the designed SAW resonator.

Figure 7 shows the measured and simulated admittance Y_11_ curves of the SAW device. The displacement contour is also given in the Figure as an insert. As shown, a transverse polarization state can be observed, which is the so-called SH wave acoustic mode. Analysis of Figure 7 indicates that the bandwidth of the measured results is consistent with the simulated bandwidth, while there is a certain discrepancy between the measured and simulated *k*^2^ values. This discrepancy arises from several factors: (1) the simulation calculations use ideal parameters, while actual materials may differ; (2) there may be discrepancies between the actual and simulated material thicknesses due to process errors and possible damage to the material lattice during production; and (3) there are electrical parasitic effects such as insertion loss.

**Figure 8:**
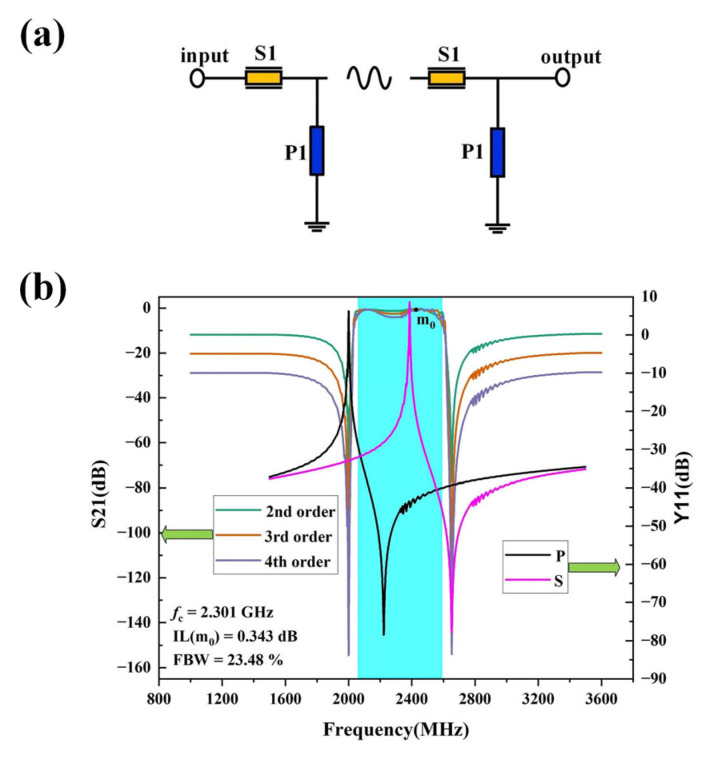
The SAW ladder filter based on the designed resonator. (**a**) The schematic of topological structure, (**b**) the simulated S parameter.

A filter prototype is implemented based on the designed resonator. Figure 8 shows the schematic of topological structure of the SAW filter and the simulated S parameter. In this case, the ladder filter consisting of series and parallel resonators with different order is simulated. The green curve here represents the simulation results of the 2nd order circuit, and optimization was performed based on the 2nd order circuit simulation result, as the passband of the 2nd order circuit simulation result is relatively flat. As can be seen from Figure 8a, the 3rd order circuit and the 4th order circuit are added in series based on the secondary circuit. Therefore, after increasing the circuit in series based on the 2nd order circuit. An impedance mismatch phenomenon occurs at the input port and output port, which explains the deterioration of the insertion loss within the passband. This also accounts for the more severe deterioration of the passband insertion loss in the 4th order circuit. Additionally, the presence of spurious waves in the high frequency area of the passband is due to the relatively large bandwidth of the SAW filter, causing the bulk wave of the parallel resonator to enter the passband. As shown in the Figure 8b, the passband ripple is within a range of 1dB, which could be further optimized for a certain desired SAW filter in future work.

As can be seen, the 4th order bandpass filter offers a better rejection level of better than 30 dB, meanwhile a center frequency of 2.3 GHz, a 3-dB fractional bandwidth of 23.48% and a minimum in-band insertion loss of only 0.343 dB are simultaneously achieved, which demonstrates the applicability for wideband SAW filter with improved performance.

## 5. Conclusions

This paper presents a SAW resonator on the Al/41° Y-X LiNbO_3_/SiO_2_/poly-Si/Si muti-layered structure and its design optimization is performed by using finite element software COMSOL5.5. The effects of the LiNbO_3_, SiO_2_, and Al electrode thicknesses on the SAW resonator performance have been analyzed. The structural parameters were optimized, and a resonator was fabricated to verify the design. The simulation results were found to be in good agreement with the measured results of the fabricated one. This study demonstrates the optimization of the structural parameters for the proposed SAW resonator offering high coupling up to 24% and its application to filter confirms a large 3-dB fractional bandwidth of 11.3%. Additionally, the structure presented can better meet the design and fabrication requirements of resonators with spurious free, which provides guidance for the development of wideband SAW filters with high-performance.

## Figures and Tables

**Table 1:** The material parameters of concern in the simulation.

	Symbol	LiNbO_3_	SiO_2_	poly-Si	Si
Elastic Constants/×10^10^N/m^2^	C_11_	19.839	7.85	18.267	16.6
	C_12_	5.472	1.61	5.15	6.4
	C_13_	6.513	1.61	5.15	6.4
	C_33_	22.79	7.85	18.267	16.6
	C_44_	5.965	3.12	6.557	79.6
Piezoelectric Constants/C/m^2^	e_15_	36.9	-	-	-
	e_31_	30	-	-	-
	e_33_	17.7	-	-	-
Relative Dielectric Constants	e_11_/e_0_	45.6	3.75	4.5	11.7
	e_33_/e_0_	26.3	3.75		
Density/kg/m^2^	*ρ*	4700	2200	2320	2329

## Data Availability

The authors have access to all the data in the study (for original research articles) and accept responsibility for its validity.
